# Excessive Iodine Promotes Pyroptosis of Thyroid Follicular Epithelial Cells in Hashimoto's Thyroiditis Through the ROS-NF-κB-NLRP3 Pathway

**DOI:** 10.3389/fendo.2019.00778

**Published:** 2019-11-20

**Authors:** Jiameng Liu, Chaoming Mao, Liyang Dong, Ping Kang, Chao Ding, Tingting Zheng, Xuefeng Wang, Yichuan Xiao

**Affiliations:** ^1^Department of Nuclear Medicine, The Affiliated Hospital of Jiangsu University, Zhenjiang, China; ^2^Key Laboratory of Stem Cell Biology, Institute of Health Sciences, Shanghai Institutes for Biological Sciences, Chinese Academy of Sciences and Shanghai Jiao Tong University School of Medicine, Shanghai, China

**Keywords:** excessive iodine, Hashimoto's thyroiditis, pyroptosis, reactive oxygen species, NLRP3 inflammasome

## Abstract

Hashimoto's thyroiditis (HT) is a common autoimmune thyroid disease. In recent years, increasing evidence has proven that the incidence of HT is associated with the excessive iodine intake of the body. In the present study, we measured the status of pyroptosis in thyroid tissues from patients with HT and the effects of excessive iodine on the pyroptosis in thyroid follicular cells (TFCs), in an attempt to illuminate the effects of iodine excess on the development of HT disease. Our results showed that increased pyroptosis occurred in the thyroid tissues of HT patients and that an increase in pyroptosis activity in TFCs was primed by excessive iodine *in vitro*. This process was mediated by reactive oxygen species (ROS) and activation of the NF-κB signaling pathway. In addition, excessive iodine caused NLRP3 inflammasome activation in TFCs, which promoted TFC pyroptosis. Moreover, the release of interleukin-1β (IL-1β) was closely linked to pyroptosis activation. Taken together, our results suggested that excessive iodine contributed to aberrant activation of pyroptosis in TFCs, which could be a pivotal predisposing factor for HT development.

## Introduction

Hashimoto's thyroiditis (HT) is a chronic form of autoimmune thyroiditis, and autoimmune hypothyroidism, lymphocytic infiltration into the thyroid tissue, and the production of antithyroid antibodies are the main manifestations of chronic inflammation of the thyroid gland in HT (1, 2). An epidemiological investigation suggests that genetic susceptibility and environmental triggers are the key factors that result in the breakdown of tolerance and the development of disease (3). The major risk factors that increase subclinical hypothyroidism and autoimmune thyroiditis include radiation, infections, stress, drugs, and excessive iodine (4). Excessive iodine intake is the main risk factor of HT. In recent years, several observational studies have reported that excessive iodine intake in different populations leads to an increase in the incidence and prevalence of HT (5). However, the specific mechanism linking excessive iodine intake and Hashimoto thyroiditis is unclear.

Iodine is essential for thyroid hormone synthesis and is a component of the thyroid hormones thyroxine (T4, prohormone) and tri-iodothyronine (T3, active hormone) (6); either low or high iodine intake may lead to thyroid diseases, such as hypothyroidism, subclinical hypothyroidism, and autoimmune thyroiditis (7, 8). It has been reported that excessive iodine may be associated with the production of antibodies specific to thyroid hormones, which also increases the production of thyroid-infiltrating T helper 17 cells (9–11). Excessive iodine induces thyroid follicular cell (TFC) injury, apoptosis, and necrosis, as well as the production of unbalanced reactive oxygen species (ROS), in the NOD-H-2^h4^ mouse model, and thus induces autoimmune thyroiditis via ROS (12). Although several studies have revealed that excessive iodine primes the accumulation of ROS, the effects of ROS in HT need to be explored further.

Pyroptosis is an inflammatory form of regulated cell death that relies on the activation of cytosolic inflammasomes (13). Previous studies have demonstrated that aberrant pyroptosis occurs in many autoimmune diseases, such as systemic lupus erythematosus (SLE), rheumatoid arthritis (RA), and Sjögren's syndrome (SS) (14–16). Many studies have identified gasdermin D (GSDMD) as an executor of pyroptosis (17–19), and one of the upstream activators of pyroptosis are inflammasome-signaling pathway components, such as nucleotide oligomerization domain (NOD)-like receptors (NLRs) (20). The NLRP3 inflammasome can be activated in response to infection or sterile inflammation mediated by endogenous DAMPs and ROS (21). The key process in pyroptotic cell death is the formation of pores, and the cleavage of GSDMD by inflammatory caspases allows its N-terminal domain (GSDMD-N) to associate with membrane lipids and form pores (22, 23). Then, GSDMD-N pores lead to cell swelling, eventual plasma membrane rupture, and the release of interleukin-1β, which in turn recruits more immune cells to promote inflammation (24, 25). However, it is not clear whether pyroptosis participates in the process of excessive iodine-induced HT.

Together, these studies have reported that inflammasomes and aberrant pyroptosis are associated with HT pathogenesis (26); however, the relationships among these factors and the mechanism of this disease are not clear. In the present study, we found aberrant pyroptosis in the thyroid tissues of HT and excessive iodine-primed pyroptosis activation through ROS-NF-κB-NLRP3 signaling in TFCs.

## Materials and Methods

### Cell Lines and Samples

The thyroid follicular epithelial cell line Nthy-ori 3-1 from the European Collection of Animal Cell Cultures (ECACC) was cultivated in RPMI-1640 (BI, USA) supplemented with 10% fetal bovine serum (BI, USA) and 2 mM L-glutamine (Biological Industries, Kibbutz Beit Haemek, Israel) in the presence of 5% CO_2_ at 37°C. Thyroid tissue glands were obtained from 20 patients with HT and 10 patients with a simple thyroid goiter as controls; those tissues were collected from the Hospital Affiliated of Jiangsu University. The HT diagnosis was based on clinical evaluations and Japanese guidelines, as described previously (27). A simple thyroid goiter diagnosis was performed according to clinical diagnosis and laboratory examination.

### Ethics Statement

This study was approved by the Ethics Committee of the Affiliated Hospital of Jiangsu University and performed in accordance with the guidelines of the Declaration of Helsinki. All thyroid samples were acquired in accordance with the regulations and approval of the Institutional Review Board of the Affiliated Hospital of Jiangsu University. Written informed consent forms were obtained from all patients.

### Reagents and Antibodies

Primary antibodies, such as rabbit anti-NFκB p65, rabbit anti-*p*-NFκB p65 (Ser536), and rabbit anti-β-actin antibodies, were obtained from Cell Signaling Technology (USA). Anti-GSDMDC1 (64-Y), anti-IL-1β, anti-Hsp60, HRP-labeled goat anti-rabbit IgG, and HRP-labeled goat anti-mouse IgG were purchased from Santa Cruz Biotechnology (USA). The anti-NLRP3 antibody was acquired from Abcam (Cambridge, UK). The IL-1β Quantikine ELISA kit was from MultiSciences (China). The ROS assay kit, N-acetyl-cysteine (NAC) and Cell Counting Kit-8 (CCK-8) were from Beyotime Biotech (China). IKK-16 was purchased from Selleck Chemicals (USA). Sodium iodide (NaI) was purchased from Sigma-Aldrich.

### IHC Staining

Thyroid samples were fixed in 10% neutralized formalin, embedded in paraffin, cut into 4-mm sections, and mounted on slides. After deparaffinization and rehydration, antigen retrieval was performed by boiling the samples in 10 mM citrate buffer (pH 6.0) for 10 min and then washing the sections with phosphate-buffered saline (PBS). Thyroid sections were blocked with 2% bovine serum albumin in PBS for 30 min and then incubated with mouse anti-human GSDMD or mouse anti-human IL-1β antibodies (Santa Cruz, NJ, USA) at 4°C overnight. After three washes with PBS, the sections were treated with the corresponding streptavidin peroxidase-coupled secondary antibody (Maixin Biotechnology Co, Ltd.). Thyroid tissue sections were then counterstained with 3,3′-diaminobenzidine and hematoxylin and observed and photographed under an optical microscope. The results of the quantitative analyses of all sections obtained with Image Pro plus 6.0 software (Version X; Media Cybernetics, USA) are presented as graphs.

### Immunoblot Analysis

Total protein was extracted from Nthy-ori 3-1 cells with RIPA (50 mM Tris-HCL, pH 7.4, 150 mM N-acetyl-cysteine l, 1% NP-40, 0.5% Na-deoxycholate, 1 mM EDTA). The protein concentration was determined using a BCA protein concentration kit (Beyotime, Shanghai, China). First, the protein extracts were subjected to electrophoresis on 10–15% acrylamide gels by SDS-PAGE and then transferred onto a polyvinylidene difluoride (PVDF) membrane (Merck Millipore, Billerica, MA, USA). After blocking for 1 h in 5% skim milk powder, the membranes were incubated with antibodies specific for target proteins, Hsp60 or β-actin (standard controls), followed by HRP-conjugated secondary antibodies. The signals were detected using the Pierce ECL-plus substrate (Thermo Fisher Scientific, Waltham, MA, USA) and scanned with the ChemiScope series (Clinx, Shanghai, China). Images were also analyzed using Clinx Image Analysis (Clinx, Shanghai, China), and the results of quantitative analyses are presented as graphs.

### Cell Viability Assay

After Nthy-ori 3-1 cells were treated with NaI for 24 h, 10 μL of CCK-8 solution was added to each well and incubated for 2 h at 37°C with 5% CO_2_. Cell viability was analyzed by a microplate reader (Bio-Rad Laboratories, Hercules, CA) at a wavelength of 450 nm.

### Detection of ROS

The accumulation of ROS in cells was detected by a fluorescent probe, 2,7-dichlorohydrofluorescein diacetate (DCFH-DA) (Beyotime, Shanghai, China), according to the manufacturer's instructions. Briefly, Nthy-ori 3-1 cells (2.5 × 10^5^ cells per well) were cultured on coverslips in six-well plates, treated with NaI (0, 20, 50 mM) and/or NAC (10 mM) for 24 h and then stained with DCFH-DA (10 μM) in pure RPMI-1640 in the dark at 37°C for 30 min, followed by three washes with pure RPMI-1640. The DCF fluorescence of 2 × 10^5^ cells was detected and analyzed immediately by flow cytometry (FACS Calibur; BD Biosciences, USA) with an excitation wavelength of 488 nm and an emission wavelength of 525 nm. The ROS levels in Nthy-ori 3-1 cells are shown as the percentage of positive cells (% positive cells) and the mean fluorescence intensity (MFI). Additionally, the ROS levels were also imaged by fluorescence microscopy (Olympus, Japan), and green fluorescence represented the amount of ROS.

### SiRNA Preparation and Transfection

Small interfering RNA molecules targeting NLRP3 were designed and synthesized by GenePharma (Shanghai, China). Nthy-ori 3-1 cells were plated in six-well plates at 3 × 10^5^ cells/well, grown for 12 h and then transfected with siRNA at a concentration of 100 nM using Lipofectamine 2000 (Invitrogen Life Technologies, Inc., Carlsbad, CA) according to the manufacturer's protocol. NLRP3 protein expression was examined at 48 h after transfection by immunoblots.

### Enzyme-Linked Immunosorbent Assay (ELISA)

Supernatant IL-1β levels were measured by a quantitative enzyme immunoassay technique with the IL-1β Quantikine ELISA Kit according to the manufacturer's instructions (MultiSciences, China) after treatment with NaI (0, 50 mM), with or without NAC (10 mM), IKK-16 (2 μM) and silencing of NLRP3, for 24 h. A microplate reader capable of measuring absorbance at 450 nm and 630 nm was used to measure the color intensity that developed in each well. All assays were performed in duplicate. The detection limit of the assay was 0.15 pg/mL.

### Statistical Analyses

The data are expressed as the mean ± SEM. GraphPad Prism 5.0 software (GraphPad Software, Inc., San Diego, CA, USA) was used to process the data. For comparisons between two groups, an unpaired *t*-test was performed; for comparisons among multiple groups, a one-way analysis of variance (ANOVA) with the Tukey–Kramer multiple comparison test was performed. *P* < 0.05 was considered statistically significant.

## Results

### Aberrant Pyroptosis Occurs in the Thyroid Tissues of HT Patients

**Figure 1:**
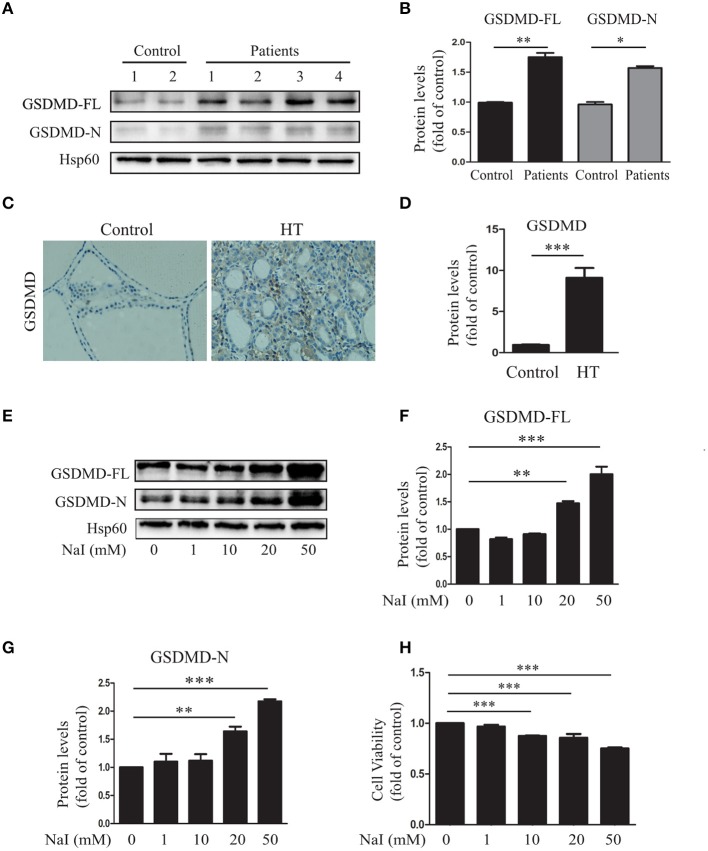
Aberrant pyroptosis occurs in the thyroid tissues of HT patients and is induced by excessive iodine *in vitro*. **(A,B)** Gasdermin D (GSDMD) protein levels in the thyroid tissue of HT patients (*n* = 20) and controls (*n* = 10) were analyzed by immunoblots. “Control” indicates tissues from patients with nodular goiter of the thyroid. Representative immunoblotting results and quantification of GSDMD are shown. **(C,D)** Representative results of GSDMD immunohistochemical staining in HT tissues (*n* = 20) and control tissues (*n* = 10) are shown. Brown regions represent positive expression (original magnification, ×200; scale bars, 100 μm). **(E–G)** Nthy-ori3-1 cells were harvested after treatment with a gradient of concentrations of sodium iodide (NaI) for 24 h. The images presented are immunoblots probed for GSDMD (Hsp60 served as the loading control). All statistical results shown are representative of three replicates. **(H)** The cell viability of Nthy-ori 3-1 cells was assessed by CCK-8 assays after NaI treatment for 24 h. All statistical results shown are representative of three replicates. Significant differences and *P*-values were calculated by unpaired *t*-tests or one-way ANOVA. *P < 0.05, **P < 0.01, ***P < 0.001.

To determine whether aberrant pyroptosis occurred in the thyroid tissues of patients with HT, the pyroptosis-related protein GSDMD in the thyroid tissues of HT patients was first evaluated. The results showed that compared with those of the control (*n* = 10), GSDMD-FL (*P* < 0.01) and GSDMD-N (*P* < 0.05) levels were significantly increased in the thyroid tissues of HT patients (*n* = 20; Figures 1A,B), as indicated by immunoblot analysis, and the level of GSDMD expression in the thyroid tissues of HT patients (*n* = 20) was also higher than that in controls (*n* = 5; *P* < 0.001; Figures 1C,D), as indicated by IHC analysis. These findings suggested that increased pyroptosis occurred in the thyroid tissues of HT patients.

To identify the inducer responsible for the increased pyroptosis in HT, the relationship between NaI and pyroptosis in TFCs was explored *in vitro*. The results showed that when Nthy-ori 3-1 cells were incubated with a gradient of concentrations of NaI (0, 1, 10, 20, and 50 mM), the expression level of GSDMD was notably increased in a dose-dependent manner (Figures 1E–G). To further characterize the excessive iodine-induced pyroptosis in TFCs, we detected the effect of excessive iodine on the viability of cells by the CCK-8 assay. The results showed that cell viability was suppressed with increasing concentrations of NaI (Figure 1H). Thus, these results suggested that abnormal pyroptosis occurred in autoimmune thyroiditis patients and was associated with excessive iodine-induced pyroptosis of TFCs.

### The ROS-NF-κB Signaling Pathway Is Involved in Excessive Iodine-Induced Pyroptosis of TFCs

**Figure 2:**
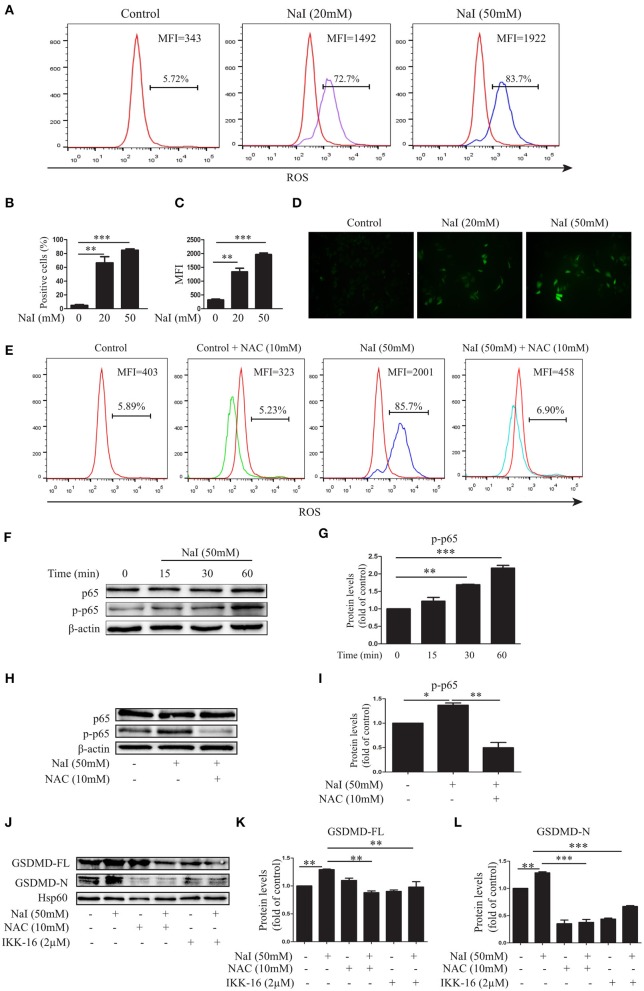
Excessive iodine-induced pyroptosis in TFCs is related to ROS-NF-κB signaling. Nthy-ori 3-1 cells were treated with NaI for 24 h. **(A–C)** The intracellular reactive oxygen species (ROS) levels were detected by flow cytometry (FCM) analysis and are presented as the positive cells (%) and mean fluorescence intensity (MFI). **(D)** The intracellular ROS levels, as indicated by DCF fluorescence, were analyzed immediately using immunofluorescence (200×; scale bars, 50 μm). **(E)** The intracellular ROS levels using NaI with or without N-acetyl-cysteine (NAC) (10 mM) treatment were detected by FCM, and representative FCM graphs of the ROS-positive cells (%) and mean fluorescence intensity (MFI) are shown. **(F,G)** Nthy-ori3-1 cells were treated with NaI at different time points, and the p65 and *p*-p65 expression levels were measured by immunoblots. **(H,I)** Nthy-ori3-1 cells were treated with NaI at 24 h in the presence or absence of NAC, and p65 and *p*-p65 expression levels were measured by immunoblots. **(J–L)** Nthy-ori 3-1 cells were treated with NaI and/or NAC and/or IKK-16 for 24 h, and GSDMD expression levels were measured by immunoblots. All statistical results shown are representative of three replicates. Significant differences and *P*-values were calculated by one-way ANOVA. *P < 0.05, **P < 0.01, ***P < 0.001.

ROS disorder plays an important role in the development of HT (28, 29). To elucidate the possible role of ROS in excessive iodine-induced pyroptosis, the fluorescent probe DCFH-DA was used to detect changes in ROS levels in TFCs under conditions of excessive iodine. Our results demonstrated that, compared with the control treatment, NaI treatment (20, 50 mM) dramatically increased ROS levels in TFCs by flow cytometry (FCM) analysis (Figures 2A–C) and fluorescence analysis (Figure 2D), and this effect was buffered by NAC (10 mM), a ROS inhibitor (Figure 2E). NAC also inhibited NaI-induced pyroptosis by decreasing GSDMD-FL and GSDMD-N levels (Figures 2J–L), suggesting that the pyroptosis activation induced by excessive iodine was partly dependent on ROS generation.

**Figure 3:**
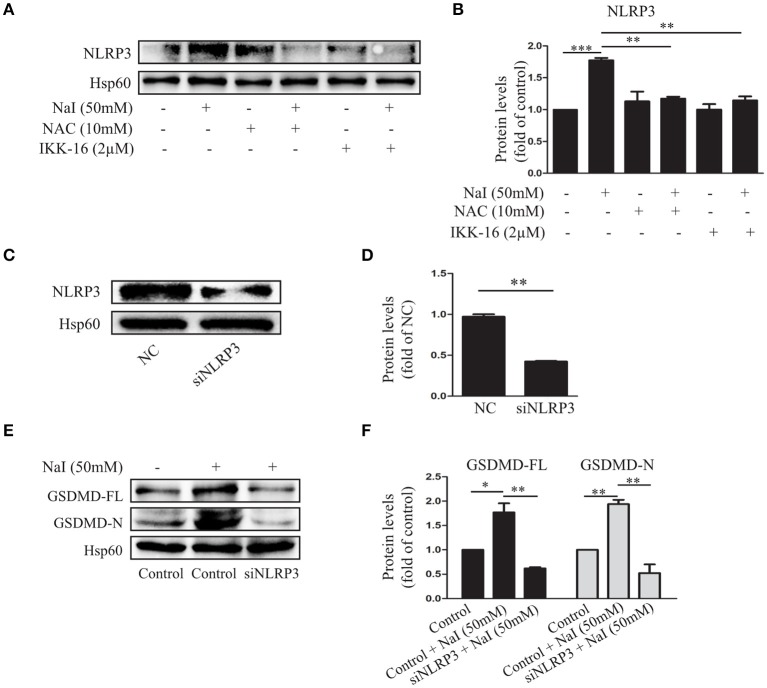
The NLRP3 inflammasome participates in excessive iodine-induced pyroptosis in TFCs. **(A,B)** The NLRP3 expression levels were measured by immunoblots when Nthy-ori 3-1 cells were treated with NaI with or without NAC (10 mM) or IKK-16 (2 μM) for 24 h. **(C,D)** Verification of the silencing efficiency of NLRP3 by siRNA in Nthy-ori3-1 cells is shown by immunoblots; NC indicates the negative control. **(E,F)** The protein levels of GSDMD were detected by immunoblots after transfection of siNLRP3 in NaI-treated Nthy-ori 3-1 cells. All statistical results shown are representative of three replicates. Significant differences and *P*-values were calculated by one-way ANOVA. *P < 0.05, **P < 0.01, ***P < 0.001.

To further determine the mechanism of excessive iodine-induced pyroptosis activation, NF-κB signaling, an important regulatory pathway of pyroptosis, was analyzed after NaI treatment in Nthy-ori 3-1 cells. The levels of phosphorylated and total p65 were examined by an immunoblot analysis. The results showed that excessive iodine induced the phosphorylation of p65 in Nthy-ori 3-1 cells (Figures 3F,G). Interestingly, the NF-κB inhibitor IKK-16 inhibited excessive iodine-induced pyroptosis by decreasing the protein levels of GSDMD-FL and GSDMD-N (Figures 2J–L), suggesting that excessive iodine induced pyroptosis activation via the NF-κB signaling pathway in TFCs. In addition, to explore the relationship between ROS and NF-κB signaling, we examined the possible changes in NF-κB signaling in the presence of NAC. The immunoblot results showed that NAC also obviously inhibited the phosphorylation of p65 under conditions of excessive iodine in Nthy-ori 3-1 cells (Figures 2H,I). Thus, these findings suggested that pyroptosis activation was dependent on the ROS-NF-κB pathway.

### The NLRP3 Inflammasome Is Involved in Excessive Iodine-Induced Pyroptosis of TFCs via ROS-NF-κB

NLRP3 inflammasomes are molecular platforms that are activated upon cellular infection or stress that triggers pyroptotic cell death (30). To further analyze the regulatory effect of NaI on inflammasome activation by NaI, we treated Nthy-ori 3-1 cells with NaI (50 mM) and examined inflammasome activity. As expected, NaI treatment stimulated the upregulation of the NLRP3 inflammasome. In contrast, Nthy-ori 3-1 cells treated with NAC (10 mM) or IKK-16 (2 μM) showed the opposite results: the NLRP3 inflammasome was suppressed (Figures 3A,B).

To further validate the participation of NLRP3 inflammasome activation in excessive iodine-induced cell pyroptosis, we assessed whether NLRP3 siRNA could affect the pyroptosis of TFCs. An immunoblot analysis indicated that pyroptosis was inhibited by GSDMD-FL, and GSDMD-N protein levels decreased when NLRP3 inflammasomes were silenced under conditions of excessive iodine (50 mM; Figures 3C–F). Thus, these findings indicated that the ROS-NF-κB pathway and NLRP3 activation executed excessive iodine-induced pyroptosis in TFCs.

### Pyroptosis Activation Contributes to IL-1β Secretion in TFCs

**Figure 4:**
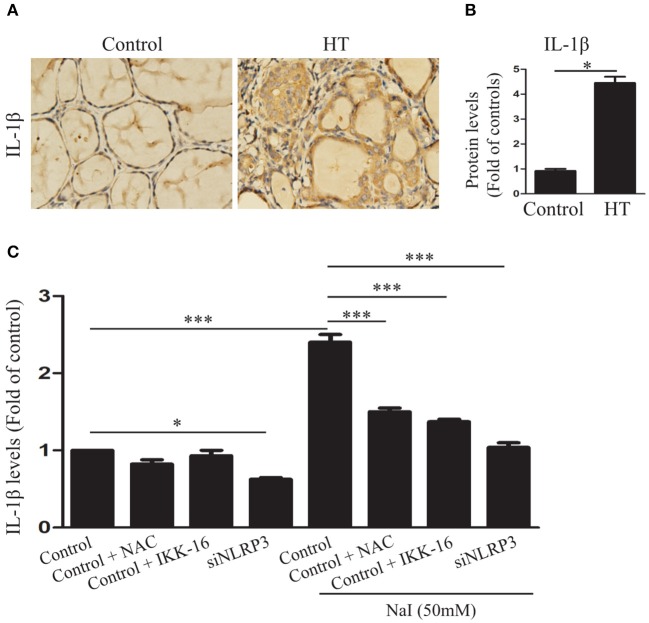
Excessive iodine-induced pyroptosis activation contributes to IL-1β secretion in TFCs. **(A,B)** Representative results of interleukin-1β (IL-1β) immunohistochemical staining in HT tissues (*n* = 20) and control tissues (*n* = 10) are shown. Brown regions represent positive expression (original magnification, ×200; scale bars, 100 μm). **(C)** IL-1β levels in the supernatants of Nthy-ori 3-1 cell cultures were detected by ELISA in the presence of NaI treatment, with or without NAC, IKK-16, and silencing of NLRP3 at 24 h. The statistical results shown are representative of three replicates. Significant differences and *P*-values were calculated by unpaired *t*-tests or one-way ANOVA. *P < 0.05, ***P <0.001.

Interleukin-1β is a major mediator of immune and inflammatory responses and plays a role in autoimmune and autoinflammatory diseases (25). To determine whether IL-1β was increased in the thyroid tissues of HT, IL-1β in the thyroid tissues of HT patients was evaluated. The results showed that, compared with the control (*n* = 10), IL-1β (*P* < 0.01) was significantly increased in the thyroid tissues of HT patients (*n* = 20; Figures 4A,B), as indicated by the IHC analysis. To further determine whether the release of IL-1β in HT is related to pyroptosis activation, we detected IL-1β in the cell culture medium by ELISA in the presence of 50 mM NaI, with or without NAC, IKK-16, and silencing of NLRP3. The study showed that, compared with control, the IL-1β concentration was dramatically increased after NaI treatment in TFCs and was reduced by the addition of NAC or IKK-16. Similarly, knockdown of the NLRP3 inflammasome also dramatically suppressed the release of IL-1β (Figure 4C). These results provided evidence that excessive iodine-induced pyroptosis promoted IL-1β release by the NLRP3 inflammasome.

## Discussion

**Figure 5:**
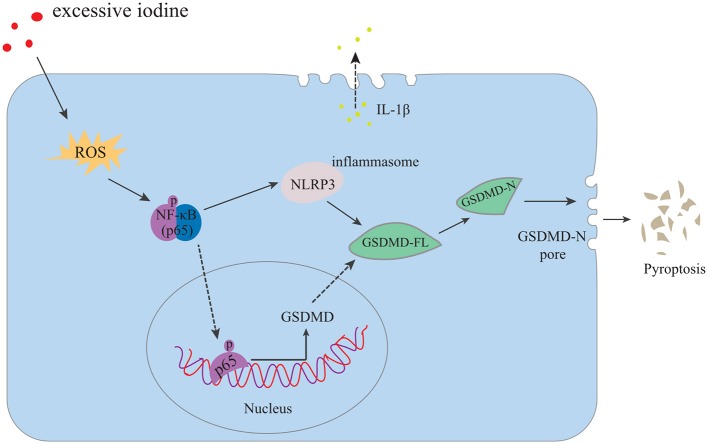
Proposed model of TFC pyroptosis in excessive iodine-promoted HT. Excessive iodine enters TFCs and induces the production of ROS, which activates NF-κB signaling and increases GSDMD-FL. In addition, the NLRP3 inflammasome is activated by NF-κB signaling, leading to GSDMD-FL cleavage into GSDMD-N, which triggers pore formation in the membrane and the release of mature IL-1β from cells, causing a sterile inflammatory response and further contributing to pyroptotic cell death and the subsequent promotion of HT.

HT is a chronic inflammatory disorder of the thyroid gland, and excessive iodine intake is the main risk factor of HT. The specific mechanisms, however, by which excessive iodine triggers autoimmune thyroiditis are still unclear (31). The data presented in the current study demonstrated that excessive iodine induced ROS accumulation, followed by NF-κB signaling, NLRP3 inflammasome activation, and subsequent abnormal pyroptosis in the setting of HT. Finally, IL-1β was released into the extracellular matrix. Reducing ROS production, suppressing NF-κB signaling or silencing the NLRP3 inflammasome inhibited NaI-induced pyroptotic death of TFCs and IL-1β secretion (Figure 5). Our study revealed aberrant pyroptotic cell death as a cellular mechanism in HT, thereby advancing our understanding of the pathophysiology of HT.

Pyroptosis is a novel and critical pathway that leads to programmed cell death in a number of settings (32, 33). In general, cell pyroptosis participates in a normal physiological pathway that maintains cellular metabolic processes, though the aberrant activation of pyroptosis can become detrimental in the context of chronic autoinflammatory diseases and sepsis (34–36). Consistent with this opinion, in this study, we found that the levels of pyroptosis in HT were abnormally increased, though they were not obvious in control thyroid tissues. Excessive iodine intake is the main risk factor of HT; however, the relationship between iodine and pyroptosis in TFCs remains unclear. In this study, we found that excessive iodine could dramatically prime pyroptosis activation in TFCs *in vitro*. Low levels of iodine, however, were not enough, which correlated to the increased levels of pyroptosis in HT and low content in the thyroid without HT. These results provide further evidence that excessive iodine acts as a pivotal pathogenic factor in HT via the pyroptosis pathway. Recently, it has been reported that GSDMD-N expression levels—but not GSDMD-FL levels—were increased in LPS-induced TFCs, which is inconsistent with our study (26). However, in mouse adipose tissue, the GSDMD-FL levels are increased by LPS treatment through the NF-κB signaling pathway (37), consistent with our study. These results suggested that pyroptosis may be a new form of programmed cell death for damaged TFCs in HT under the condition of excessive iodine.

The accumulation of ROS has been reported in many diseases related to pyroptosis (38–40). On the other hand, the NF-κB signaling pathway is significantly enriched in the inflammatory response, and many studies have demonstrated that ROS can activate the NF-κB signaling pathway (41). In agreement with this view, our research showed that excessive iodine promoted ROS production, and this oxidative stress is an upstream mechanism for the activation of pyroptosis through the NF-κB p65 signaling pathway in TFCs. Our previous studies have reported that an increase in ROS results in autophagy and apoptosis of TFCs in autoimmune thyroid disease (28, 29). Hence, our research provides a new factor in the pathogenesis of ROS in HT: ROS increased pyroptosis in HT.

Activation of pyroptosis requires a protein platform called the inflammasome. Among the inflammasomes, the NLRP3 inflammasome is the most extensively studied (42). The NLRP3 inflammasome can be activated upon exposure to whole pathogens, as well as a number of structurally diverse PAMPs, DAMPs, and environmental irritants (43). The fact that there are strong associations between dysregulated inflammasome activity and human heritable and acquired inflammatory diseases emphasizes the importance of this pathway in immune responses (30). It has been reported that the NLRP3 inflammasome plays an important role in inflammation-associated atherosclerosis and obesity (34, 44), and a study showed that NLRP3 inflammasome levels are increased in HT (26), but the specific mechanism in HT is not clear. We found here that the expression of the NLRP3 inflammasome was upregulated via the ROS-NF-κB pathway in excessive iodine-treated TFCs. In contrast, after silencing the NLRP3 inflammasome in TFCs in small interfering RNA experiments, these cells became immune to NaI-induced pyroptotic death, suggesting that the NLRP3 inflammasome is required for excessive iodine-induced pyroptosis in TFCs.

Interleukin-1β (IL-1β) is one of the key proinflammatory cytokines in the interleukin-1 (IL-1) family that plays an important role in innate immune responses and is produced by monocytes, endothelial cells, and other types of cells in response to stimulation (45). It has been reported that IL-1β is a proinflammatory cytokine that participates in the abnormal proliferation of TFCs, leading to the destruction of thyroid tissue during HT pathogenesis (46). In the current study, our results demonstrated that IL-1β levels were increased in the thyroid tissues in HT and induced in TFCs by excessive iodine, suggesting that IL-1β plays a vital role in HT pathogenesis. In addition, it has been reported that IL-1β release is based on inflammasome activation and pyroptosis in macrophages (24), in agreement with our finding that the secretion of mature, active IL-1β into the extracellular matrix was dependent on pyroptosis in HT.

However, this study has some limitations. For example, our results were determined only in a TFC line and need to be confirmed in other thyroid follicular epithelial cells. Moreover, this study mainly focused on *in vitro* experiments, and whether animal experimentation will yield the same results needs to be explored. Our results provide the first evidence that the pyroptosis mechanism in HT is likely a cellular mechanism that underlies the detrimental effect of excessive iodine through the production of ROS and activation of the NLRP3 inflammasome as upstream mediators. In general, iodine is necessary to maintain the normal function of thyroid tissue, and iodine deficiency is associated with developmental delays, endemic goiter, and other issues (47). Moderate iodine fortification is an effective method to prevent thyroid disorders, reducing the long-term risk of overt thyrotoxicosis without increasing hypothyroidism (48). However, it is also noted that the downside of excessive iodine intake was a detrimental effect on thyroid tissues, as the present study and numerous studies reported (7, 28).

## Data Availability Statement

All datasets generated for this study are included in the article/supplementary material.

## Ethics Statement

The studies involving human participants were reviewed and approved by Biomedical research ethics committee, the Affiliated Hospital of Jiangsu University. The patients/participants provided their written informed consent to participate in this study. Written informed consent was obtained from the individual(s) for the publication of any potentially identifiable images or data included in this article.

## Author Contributions

JL performed most of the experiments, analyzed the data, and wrote the manuscript. CM and LD designed the project, evaluated and interpreted the data, wrote the manuscript, and financed and supervised the study. TZ edited various parts of the manuscript. PK and CD performed some of the experiments and evaluated the data. YX and XW supervised the data analysis and edited the manuscript.

### Conflict of Interest

The authors declare that the research was conducted in the absence of any commercial or financial relationships that could be construed as a potential conflict of interest.
